# Treosulfan-Based Conditioning Regimen for Second Allograft in Patients with Myelofibrosis

**DOI:** 10.3390/cancers12113098

**Published:** 2020-10-23

**Authors:** Isik Kaygusuz Atagunduz, Evgeny Klyuchnikov, Christine Wolschke, Dietlinde Janson, Silke Heidenreich, Maximilian Christopeit, Francis Ayuk, Nicolaus Kröger

**Affiliations:** 1 Hematology Department, University Medical Center Hamburg-Eppendorf, 20251 Hamburg, Germany; isik.kaygusuz@marmara.edu.tr (I.K.A.); eklyuchnikov@uke.de (E.K.); wolschke@uke.de (C.W.); d.janson@uke.de (D.J.); s.heidenreich@uke.de (S.H.); m.christopeit@uke.de (M.C.); ayuketang@uke.de (F.A.); 2 Hematology Department, Marmara University Pendik Training and Research Hospital, 34899 Istanbul, Turkey

**Keywords:** treosulfan, myelofibrosis, second allogeneic transplant, stem cell transplantation

## Abstract

**Simple Summary:**

Currently, the only curative therapy in myelofibrosis is allogeneic hematopoietic stem cell transplantation. Donor lymphocyte infusion and second stem cell transplantation are the two main treatment options for myelofibrosis patients who relapse after the first transplantation. The optimal conditioning regimen for the second transplantation in myelofibrosis patients is not well defined. Our study aimed to address this question and showed that treosulfan-based conditioning for second allograft in relapsed myelofibrosis patients resulted in longtime freedom from disease in about 50% of the patients. This data supports the second allogeneic hematopoietic stem cell transplantation with a less toxic treosulfan-based conditioning regimen that is effective in relapsed, donor lymphocyte infusion resistant myelofibrosis patients with long term low transplant-related mortality and relapse rates.

**Abstract:**

Relapse after allogeneic hematopoietic stem cell transplantation (AHSCT) in myelofibrosis (MF) patients remains as a significant issue despite advances in transplantation procedures and significant prolongation in survival. Second AHSCT is a potential treatment option but associated with high treatment-related mortality and novel less toxic conditioning regimens are needed. In 33 MF patients with relapse after AHSCT and failure to donor lymphocyte infusion (DLI) we investigated treosulfan (36–42 g/m^2^) in combination with fludarabine and anti-thymocyte globulin (ATG) as conditioning regimen for a second AHSCT with matched related (*n* = 2), unrelated (*n* = 23), or mismatched unrelated (*n* = 8) donors. All patients achieved leukocyte engraftment after a median of 11 days, and 56 ± 13% experienced acute GVHD grade II–IV at day 100. The therapy-related mortality at day 100 and at 3 years was 16% and 31%, respectively. The cumulative incidence of relapse at 5 years was 16%, resulting in a 5-year disease-free and overall survival of 45% and 47%, respectively. Treosulfan-based conditioning for second allograft in relapsed MF patients resulted in about 50% of the patients in long-term freedom from disease.

## 1. Introduction

Myelofibrosis (MF) is a clonal hematopoietic stem cell disorder characterized by clonal ineffective hematopoiesis, a reactive reticulin deposition and fibrosis in bone marrow, circulating CD34^+^ progenitor cells, extramedullary hematopoiesis, and leukemic progression [1]. Allogeneic hematopoietic stem cell transplantation (AHSCT) is the only potentially curative treatment option for patients with MF with a cure rate of 30–65% [2,3,4].

Prospective and retrospective studies reported an overall survival (OS) rate of 30–60% at 3–5 years for AHSCT with myeloablative conditioning (MAC) regimen in patients with MF and a non-relapse mortality (NRM) rate ranging from 30% to 48% at 1 year, 24% to 43% at 3–5 years [2,3,4,5]. Over the past decades, the use of reduced-intensity conditioning (RIC) has lowered the rate of NRM in comparison with MAC [6,7,8].

Relapse is one of the major causes of treatment failure after AHSCT in MF patients. Clinical relapses can be treated preferentially by donor lymphocyte infusion (DLI) and/or with a second AHSCT [5,9] in the presence of limited information on the efficacy and safety of the second AHSCT driven from case reports and studies with small patient numbers [9,10,11]. The preferred conditioning regimens for myelofibrosis are either busulfan or melphalan based [4,7,8]. The optimal conditioning regimen for the second AHSCT in MF patients that should provide a lower treatment-related mortality with high anti-malignancy efficacy is not well defined. Due to intense previous treatment regimens and infection-related complications, the treatment related mortality (TRM) and relapse rates may be expected to be high in these high-risk patients.

Treosulfan is a water-soluble, bifunctional, alkylating drug that shows strong myelotoxic, immunosuppressive properties, and is effective against a variety of human tumor cell lines including hematologic malignancies such as leukemia, lymphoma, and myeloma [12]. Preclinical studies showed that treosulfan has a myeloablative effect on committed and noncommitted stem cells and possesses potent immunosuppressive characteristics as the prodrug of a alkylating cytotoxic agent [13]. Treosulfan and fludarabine combination has been proven to be effective in AML and MDS patients and provided full donor chimerism with less toxicity and therefore it is accepted as a myeloablative preconditioning regimen with reduced toxicity [14]. The only retrospective study on MF patients reported that a treosulfan and fludarabine based conditioning for the first AHSCT has a potent myeloablative and antidisease activity [15].

Our study aims to analyze the safety and efficacy of a treosulfan-based conditioning regimen for the second AHSCT in patients with MF.

## 2. Materials and Methods

### 2.1. Patient Selection

In this cross-sectional study, we retrospectively evaluated the safety and efficacy of treosulfan-based conditioning regimen for second AHSCT in patients with MF. We identified 33 patients with MF who received second AHSCT between November 2005 and May 2020 at the University Medical Center Hamburg-Eppendorf, Germany.

### 2.2. Patient Characteristics

A total of 33 patients were studied with a median follow-up of 28.36 months (range: 0.3–146.36). Median age at transplantation of the whole study population was 59 years (range: 37–76). Twelve patients (36%) had primary MF (PMF), eight (24%) had post essential thrombocythemia (ET) myelofibrosis, seven (21%) had post polycythemia vera (PV) myelofibrosis, and six patients had acute myeloid leukemia (transformed from primary MF in four patients and post PV-MF in two patients). All patients had received busulfan (10 mg/kg orally or 8 mg/kg IV), fludarabine (150 mg/m^2^), and anti-thymocyte globulin (ATG) as a conditioning regimen in the first AHSCT. Median time from the first transplant to relapse was 5.5 months (range: 0.4–11.8). Median time from the first transplant to the second AHSCT was 15.5 months (range: 4.1–116.9). Median time from relapse to the second AHSCT was 6.25 months (range: 1.0–52.1). Four patients did not receive any therapy before the second AHSCT. Twelve patients received only DLI (1–5 doses), one patient DLI and hydroxyurea, five patients DLI (1–5 doses) and ruxolitinib, one patient two doses of DLI and stem cell boost, six patients only ruxolitinib, one patient one dose of DLI and azacytidine and two patients one dose of DLI and chemotherapy combination. All but leukemia patients were transplanted in an active disease status. Four of the six patients with leukemia transformation from MF were transplanted with progressive disease, one with complete remission, and one in aplasia.

After excluding patients with leukemia at the time of transplantation, Dynamic International Prognostic Scoring System (DIPSS) score at transplantation was available in 22 out of 27 patients. DIPSS score was low to intermediate-1 in six patients (18.2%), and intermediate-2 to high in 16 patients (48.5%). Four patients had been splenectomized and 18 patients had splenomegaly at the time of second transplantation with a spleen size ranging between 14.5 and 29.5 cm. Spleen size was normal in seven patients, larger than 20 cm in nine patients, and the data was missing in four patients. Patients did not receive radiotherapy or undergo any specific intervention for splenomegaly before transplantation. Cytogenetic analysis was available in 11 cases and showed normal karyotype (*n* = 6), complex karyotype (*n* = 1), and other abnormalities (*n* = 4).

All patients received peripheral blood stem cells as stem cell source from an either fully human leukocyte antigen (HLA)-matched related donor (MRD, *n* = 2), matched unrelated donors (MUD, *n* = 23), or mismatched unrelated donors (MMUD, *n* = 8). Mismatched donors had at least one allele or antigen mismatch: A locus, *n* = 3, B locus, *n* = 1, C locus, *n* = 1, DRB1 locus, *n* = 2, locus B plus C, *n* = 1. The median number of transplanted CD34^+^ cells/kg BW was 8.0 × 10^6^ (range: 2.56–15.7). Twenty-four patients had a positive cytomegalovirus (CMV) status before transplantation, and nine patients had a negative CMV serostatus at the time of transplantation. Patient characteristics are shown in Table 1.

### 2.3. Donor Characteristics

The median age was 40 years (range: 20–66) of donors (M/F = 26/7). Six male patients received a graft from a female donor. Four patients were transplanted from the same donor of the first transplant and the remaining 29 had an alternative new donor.

### 2.4. Therapy Plan

Conditioning regimen consisted of treosulfan (36–42 g/m^2^, given in daily single doses on day-7 to -5 in a dose of 12–14 g/m^2^: 36 g/m^2^: *n* = 32 and 42 g/m^2^: *n* = 1), fludarabine (150 mg/m^2^ intravenously (i.v.) given divided from day-7 to -3), anti-thymocyte globulin (ATG; rabbit, Fresenius, Bad Homburg, Germany, *n* = 6), given at a dose of 30–60 mg/kg or thymoglobulin (Genzyme GmbH, Neu-Isenburg, Germany, *n* = 26) given at a dose of 4–8 mg/kg, followed by AHSCT on day 0. Patients with leukemic transformation received fludarabine, amsacrine, cytosine arabinoside (FLAMSA) (*n* = 4), gemtuzumab, mitoxantrone and cytosine arabinoside (*n* = 1), or thiotepa (*n* = 1) with treosulfan.

### 2.5. Supportive Care

The graft-versus-host disease (GvHD) prophylaxis consisted of cyclosporine A (CSA) and mycophenolate mofetil (MMF) in 26 patients, CSA and methotrexate in four patients, and tacrolimus and MMF in three patients. Standard criteria were used for grading of acute and chronic GvHD [16,17]. Immunosuppressive drugs doses, growth factor, antibacterial, antiviral, antifungal prophylaxis, and infection treatments were performed in accordance with the center’s follow-up protocols [18].

### 2.6. Statistical Analysis

Statistical analysis of demographic variables was expressed descriptively. Data distribution was assessed using the Kruskal–Wallis test. We used Student’s *t*-test to compare continuous data with the normal distribution. The survival data were estimated using the Kaplan Meier method and log-rank test, respectively. Overall survival (OS) was calculated from the date of AHSCT until death or last observation alive. Disease-free survival (DFS) was defined as the time from AHSCT to death or relapse whichever came first. Estimation of relapse incidence and treatment-related mortality incidence was carried out using the proper estimation of the cumulative incidence curve.

## 3. Results

### 3.1. Engraftment

The median time until leukocyte (>0.5 × 10^9^/L) and platelet (>20 × 10^9^/L) engraftment was 11 days (range: 9–78) and 14 days (range: 9–119), respectively (Table 2). One patient died before engraftment at day nine of AHSCT. Except this patient, all patients achieved leukocyte engraftment and 27 out of 32 patients achieved thrombocyte engraftment.

All patients but one had complete data for engraftment. Only one patient died before the engraftment. Primary graft failure was observed only in one of 32 patients. This patient died 16 months after AHSCT due to aspergillus pneumonia and acute respiratory distress syndrome (ARDS). None of the patients developed secondary graft failure. Chimerism analysis was available in 28 out of 33 patients. All of 28 patients reached full donor chimerism at a median of 99 days (range: 19–1632). Twenty of 26 patients achieved full donor chimerism at the 30th day of AHSCT and 18 out of these 20 patients maintained their full donor chimerism status. Twenty-one of 28 patients who achieved full donor chimerism at any time point after AHSCT maintained their full donor chimerism status.

### 3.2. Graft-versus-Host Disease

At day 100, the cumulative incidence of acute GvHD in any grade, grade II–IV acute GvHD and grade III–IV acute GvHD was 73 ± 10%, 56 ± 13% and 33 ± 13%, respectively. One year cumulative incidence of moderate to severe chronic GvHD was 28 ± 12%. Median time to acute and chronic GvHD in all grades were 18.5 days (range: 9–137) and 197 days (range: 33–1412), respectively.

### 3.3. Response

Maximum response was evaluated in only 27 patients due to missing data. Overall response rate (ORR) was 85%. Nineteen patients (70%) achieved molecular complete response, two patients achieved complete histo-hematological response, two had partial responses, and the remaining patients had stable disease. The median time to maximum response was 90 days (range: 17–882).

### 3.4. Toxicity and TRM

Mucositis (*n* = 20), liver (*n* = 8), kidney (*n* = 4), gastrointestinal system (*n* = 1) toxicities developed during follow up. Twenty-seven out of 33 patients experienced an infection in the first 30 days of AHSCT. CMV reactivation developed in a total of 15 patients. In 10 of these patients, reactivation developed within the first month of AHSCT. Organ involvement (CMV—pneumonia) was observed in two of 15 patients who developed CMV reactivation. Ebstein–Barr virus (EBV) reactivation was observed in nine patients. In five of these patients EBV reactivation developed within the first month after AHSCT. Organ involvement was seen in two patients. EBV-encephalitis was detected in one patient and gastrointestinal system (GIS) involvement in one patient. The list of documented infections is given in Table 3.

Four patients experienced moderate liver veno-occlusive disease (VOD) within the first 30 days after transplant. All patients received defibrotide plus supportive therapy and VOD resolved in all.

At the time of analysis and in a median follow-up of 28.36 months (range: 0.3–146.36), 16 out of 33 patients (49%) were alive; of those, 13 (81%) were in remission.

Cause of death was not recorded for two patients, four patients died of relapse related complications and 11 of transplant related causes (GVHD:5, infection:5, GVHD and infection:1 patient). The following complications resulted in death in patients with infections: CVM pneumonia, ileitis, and intestinal perforation in one, neutropenic fever and Grade 4 GVHD-related liver failure in one, septic shock in two, mucor pneumonia in one, and aspergillus pneumonia and ARDS in one patient.

**Figure 1:**
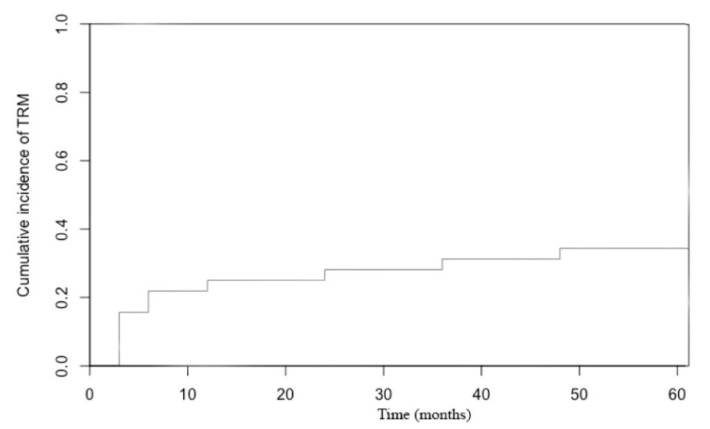
Cumulative incidence of treatment related mortality.

The cumulative incidence of TRM at day 100 was 16% (95% CI: 5.5–30.3). The 3-year TRM was 31% (95% CI: 16–47.7) and the 5-year TRM was 34% (95% CI: 18.4–50.9) (Figure 1).

### 3.5. Outcome/Overall and Disease-Free Survival

**Figure 2:**
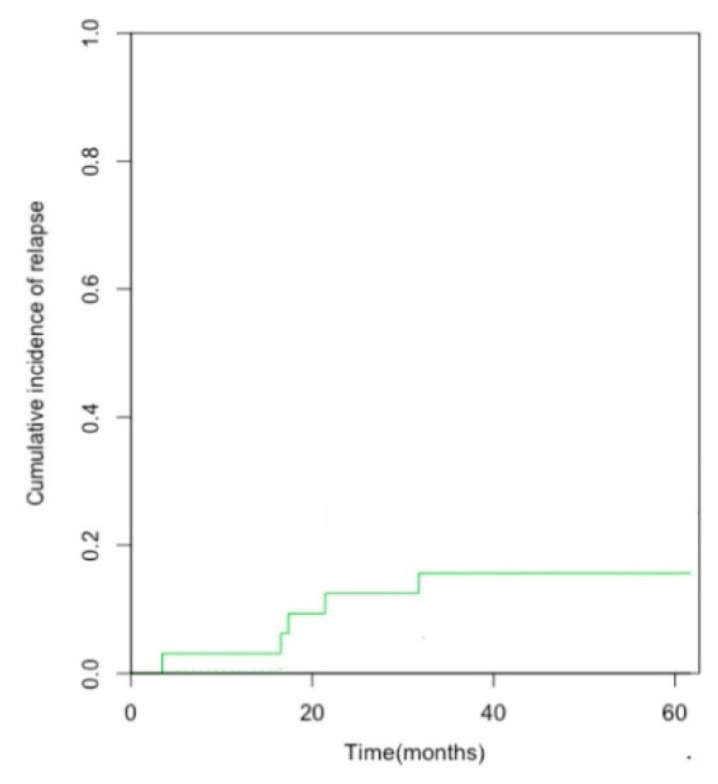
Cumulative incidence of relapse.

After a median follow-up of 21.4 months (range: 3.4–116.6), nine out of 32 patients relapsed. The 3 or 5-years cumulative incidences of relapse were both 16% (95% CI: 5.5–30.4) (Figure 2).

**Figure 3:**
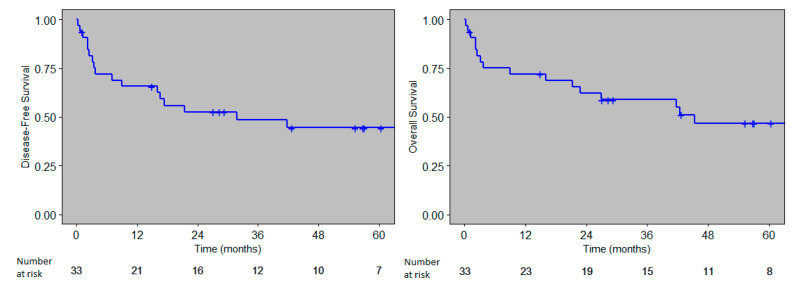
Disease free survival and overall survival.

The 3-year and 5-year estimated disease-free survival rates and overall survival rates were 49% (95% CI: 27.7–75.2)–45% (95% CI: 23.9–72.9), and 59% (95% CI: 39.1–82.02)–47% (95% CI: 27.7–75.2), respectively (Figure 3).

After excluding patients with leukemia at the time of transplantation, the 3 and 5-year OS rates for the remaining 27 patients were 62% (95% CI: 40–85) and 47% (95% CI: 25.6–78), respectively. The 3- and 5-year DFS were 49% (95% CI: 25.6–78), and 44% (95% CI: 20–75.3), respectively.

Three out of six patients diagnosed with leukemic transformation prior to transplantation died within the first 100 days after transplant due to transplant-related causes. One patient died of grade 4 acute GVHD (grade 4 acute liver GVHD-induced liver failure) and septic shock, one patient of grade 4 acute GVHD, and one patient of septic shock.

## 4. Discussion

Myelofibrosis is a clonal hematopoietic stem cell disorder. An optimal conditioning regimen for the second AHSCT in MF patients should provide low treatment-related mortality with high anti-malignancy efficacy.

The current study shows that treosulfan in combination with fludarabine as dose-reduced conditioning for patients with MF before the second AHSCT is a feasible and effective conditioning regimen.

In the current literature, knowledge on the second transplant in relapsing MF patients is sparse, and the efficacy or safety of an optimal conditioning regimen for the second transplant in these patients is not well defined. RIC regimens, fludarabine/busulfan (FB) or fludarabine/melphalan (FM) are widely used for conditioning prior to the first transplantation in MF [4,6,19,20]. The only retrospective study on MF patients reported that a treosulfan and fludarabine based conditioning for the first AHSCT has a potent myeloablative and antidisease activity [15]. There is an unmet need for novel conditioning regimens that will reduce AHSCT-related toxicity while retaining maximal antimalignancy effect [13].

To the best of our knowledge, this is the first study evaluating safety and efficacy in MF patients after the second AHSCT.

In a recent and large retrospective study of EBMT in AML, a treosulfan based conditioning regimen was similar to busulfan, at a myeloablative or a reduced dose, in achieving 2 years OS in AML patients with a possibly better safety profile in older patients, with lower rates of graft-versus-host disease and possibly better outcomes in patients with active leukemia [21]. A further prospective, phase III study of treosulfan reported that treosulfan is non-inferior to busulfan when used in combination with fludarabine as a conditioning regimen for AHSCT for older or comorbid patients with acute myeloid leukemia or myelodysplastic syndrome [14]. The improved outcomes in patients treated with the treosulfan–fludarabine regimen as a potential standard preparative regimen in this population suggest that the same regimen may be considered as effective and less toxic conditioning for MF.

Treatment of MF after the first relapse is a challenging medical condition and an optimal conditioning regimen for a more resistant MF with lower treatment-related mortality and high antimalignancy efficacy, such as treosulfan based conditioning regimens, may, therefore, be promising in these high-risk MF patients.

In the only study evaluating the effectiveness of treosulfan based conditioning regimen in the first transplant of MF, Claudiani et al. reported the outcome of 14 patients. In this group of patients with a median age of 57, full myeloablation was achieved in all patients, and full donor chimerism was achieved in 12 out of 13 patients that could be evaluated in the first month and continued in 10 out of 13 patients until a median of 39 months. Non-hematological toxicity was modest, and no patients developed VOD. After a median follow-up of 39 months (range: 2–107), the 3-year probability of OS is 54 ± 14% and the 3-year probability of DFS is 46 ± 14%. The cumulative incidence of NRM at 2 years was 39 ± 15%. Causes of NRM were infection (*n* = 2), GVHD (*n* = 2), and hemorrhage (*n* = 1). In particular, all four patients receiving a MUD SCT died because of NRM [15].

In our previous study evaluating the outcome and treatment strategies of 27 MF patients who relapsed after RIC-based transplantation, 13 of 17 patients who were treated with a second transplant with a treosulfan/fludarabine conditioning regimen and 1-year cumulative incidence of non-relapse mortality was 6%, and the cumulative incidence of relapse was 24%. These encouraging results are the first observations supporting a treosulfan based conditioning regimen in the second AHSCT in MF [9].

The importance of our present study lies in that it is the first study addressing the long-term follow-up and survival rates of a treosulfan based conditioning regimen in MF patients with relapse after the first transplant. In our study, full donor chimerism was achieved in all patients, and TRM (16% on the 100th day, 31% on the 3rd year), and relapse rates (16% on the 3rd and 5th years) were low with promising survival rates (3 yearly DFS 49%, OS 59%) better than in the previously published data [15].

Our aforementioned low TRM and relapse rates for the second AHSCT with a treosulfan based conditioning regimen are comparable with, if not better than, the TRM and relapse rates of MF patients who underwent Bu/Flu based RIC in the first AHSCT. This previous, prospective multicenter study reported the cumulative incidence of NRM at 1 year as 16%, the relapse rate at 3 years as 22% [4]. A similar, large retrospective registry analysis of the Center for International Blood and Marrow Transplant Research (CIBMTR), that included MF patients that received AHSCT with RIC regimen, the probabilities of OS at 5 years were 47% and the cumulative incidence of NRM and relapse/progression at 5 years were 24% and 48%, respectively [22].

Graft failure is one of the major complications in patients with MF undergoing AHSCT, especially after RIC regimen, with an incidence ranging from 2% to 24% [4,23]. We observed only one primary graft failure in our study. On the 30th day, full chimerism was achieved in 20 out of 26 patients, and after a median of 99 days follow-up, complete chimerism was achieved in the all 26 remaining patients.

Second AHSCT in MF is not studied in detail in the literature and only case reports and studies with small patient numbers are available [9,10,11]. We previously reported acceptable toxicity and outcomes in a cohort of relapsed patients who underwent a second AHSCT with the majority receiving fludarabine, treosulfan, and ATG-based conditioning. In this study, 13 patients with DLI failure and four patients not suitable for DLI treatment achieved 60% CR and 80% ORR with second AHSCT. Thirteen of 17 patients received treosulfan (36 g/m^2^) and fludarabine (150 mg/m^2^) as a conditioning regimen. One-year cumulative incidence of non-relapse mortality for recipients of a second allograft was 6% (95% CI: 0–18%) and the cumulative incidence of relapse was 24%, the 1-year OS and PFS for 17 patients who underwent the second AHSCT was 82% (95% CI: 58–100%) and 70% (95% CI: 35–100%), respectively [9]. These aforementioned results are in line with our current long-term follow-up data.

In conclusion, our data support a second AHSCT in relapsed, DLI resistant MF patients with a treosulfan based, effective and less toxic conditioning regimen that has long-term low transplant-related mortality and relapse rates.

## 5. Conclusions

Treosulfan-based conditioning for second allograft in relapsed myelofibrosis patients resulted in about 50% of the patients in long-term freedom from disease.

## Figures and Tables

**Table 1:** Patient characteristics.

Number of Patients	*n* = 33
Median patient age at AHSCT (years)	59 years (range: 37–76)
Median donor age at AHSCT (years)	40 years (range: 20–66)
Recipient gender	
Male/Female	*n* = 19/14
Donor gender	
Male/Female	*n* = 26/7
Male patient/female donor	*n* = 6
Diagnosis at transplantation	
Primary myelofibrosis	*n* = 12 (36 %)
Post-PV myelofibrosis	*n* = 7 (21%)
Post-ET myelofibrosis	*n* = 8 (24%)
Acute Myeloid Leukemia	*n* = 6 (18%)
DIPSS at transplantation	
Low-Int-1	*n* = 6 (18.2 %)
Int-2-High	*n* = 16 (48.5%)
Cytogenetic analysis	
Normal karyotype	*n* = 6
Complex karyotype	*n* = 1
Other abnormalities	*n* = 4
Stem cell source	
Peripheral blood stem cells	*n* = 33
Bone marrow	*n* = 0
Donor Type	
Unrelated donor	*n* = 31
Related donor	*n* = 2
Donor Status	
Same donor	*n* = 4
Alternative donor	*n* = 29
HLA-Status	
Matched	*n* = 25
Mismatched	*n* = 8
CMV Status of recipient/donor	
+/+	*n* = 23
-/-	*n* = 5
+/-	*n* = 4
-/+	*n* = 1
Median number of transplanted CD34^+^ cells/kg BW	8.0 × 10^6^ (range: 2.56–15.7)
Conditioning regimen	
Treosulfan/Fludarabine	*n* = 26
FLAMSA/Treosulfan	*n* = 4
Treo/AraC/Mitox/Gemtu	*n* = 1
Treosulfan/Fludarabine/Thiotepa	*n* = 1
Treosulfan/Thiotepa	*n* = 1
GVHD prophylaxis	
CSA + MMF	*n* = 26
CSA + Methotrexate	*n* = 4
Tacrolimus and MMF	*n* = 3

**Abbreviations:** AHSCT: allogeneic hematopoietic stem cell transplantation, DIPSS: Dynamic International Prognostic Scoring System, CSA: cyclosporine-A, MMF: mycofenolate mofetil, AraC: Cytosine arabinoside, Mitox: Mitoxantron, Gemtu: Gemtuzumab, GVHD: graft-versus-host disease. Data were presented as median (range).

**Table 2:** Results.

Median time until leukocyte engraftment (days).	11 days (range, 9–78)
Median time until thrombocyte engraftment (days)	14 days (range, 9–119)
Response Rates (*n* = 27)	
ORR mCR hCR PR	*n* = 23 (85%) *n* = 19 (70%) *n* = 2 (7%) *n* = 2 (7%)
Cumulative incidence of acute GvHD at day 100 (*n* = 33)	
All Grades Grade II-IV Grade III-IV	73 ± 10 % 56 ± 13 % 33 ± 13 %
Cumulative incidence of chronic GvHD at 1 year (*n* = 33)	
Moderate or severe	28 ± 12 %
3-year cumulative incidence of relapse	16% (CI: 5.5–30.4)
Day 100 treatment related mortality	16% (CI: 5.5–30.3)
3-year treatment related mortality	31% (CI: 16–47.7)
3-year disease-free survival	49% (CI: 27.7–75.2)
5- year disease-free survival	45% (CI: 23.9–72.9)
3-year overall survival	59% (CI: 39.1–82)
5- year overall survival	47% (CI: 27.7–75.2)

**Abbreviations:** ORR; overall response rate, mCR; molecular complete response, hCR; histo-hematological response; PR; partial response; GVHD; graft-versus-host disease.

**Table 3:** The list of infections in the follow-up of AHSCT.

Infections	Number of Patients
Pneumonia Bacterial pneumonia Viral pneumonia Fungal pneumonia (Aspergillus/Mucor) CMV-pneumonia H1N1-pneumonia	*n* = 13 *n* = 3 *n* = 2 *n* = 5 (4/1) *n* = 2 *n* = 1
Catheter related infection	*n* = 5
Blood culture positive septicemia	*n* = 9
Sepsis Catheter related sepsis Bacterial Sepsis	*n* = 7 *n* = 2 *n* = 5
BK/JCV Cystitis	*n* = 5
HSV-viremia	*n* = 1
HHV-6 infection	*n* = 1
Neutropenic Colitis	*n* = 1
